# Habituation of laser-evoked potentials by migraine phase: a blinded longitudinal study

**DOI:** 10.1186/s10194-017-0810-6

**Published:** 2017-10-02

**Authors:** Martin Uglem, Petter Moe Omland, Marit Stjern, Gøril Bruvik Gravdahl, Trond Sand

**Affiliations:** 1 Department of Neuromedicine and Movement Science, NTNU, Norwegian University of Science and Technology, Trondheim, Norway; 2 Department of Neurology and Clinical Neurophysiology, St. Olavs Hospital, Trondheim, Norway; 3 Norwegian Advisory Unit on Headaches, St. Olavs Hospital, Trondheim, Norway; 4 NTNU, Faculty of Medicine and Health Sciences, P.B. 8905, N-7491 Trondheim, Norway

**Keywords:** Headache, Migraine cycle, Pain, Pathophysiology, Preictal, Ictal, Premonitory, Laser evoked potential, Habituation, LEP

## Abstract

**Background:**

Migraineurs seem to have cyclic variations in cortical excitability in several neurophysiological modalities. Laser-evoked potentials (LEP) are of particular interest in migraine because LEP specifically targets pain pathways, and studies have reported different LEP-changes both between and during headaches. Our primary aim was to explore potential cyclic variations in LEP amplitude and habituation in more detail with a blinded longitudinal study design.

**Methods:**

We compared N1 and N2P2 amplitudes and habituation between two blocks of laser stimulations to the dorsal hand, obtained from 49 migraineurs with four sessions each. We used migraine diaries to categorize sessions as interictal (> one day from previous and to next attack), preictal (< one day before the attack), ictal or postictal (< one day after the attack). Also, we compared 29 interictal recordings from the first session to 30 controls.

**Results:**

N1 and N2P2 amplitudes and habituation did not differ between preictal, interictal and postictal phase sessions, except for a post hoc contrast that showed deficient ictal habituation of N1. Habituation is present and similar in migraineurs in the interictal phase and controls.

**Conclusions:**

Hand-evoked LEP amplitudes and habituation were mainly invariable between migraine phases, but this matter needs further study. Because hand-evoked LEP-habituation was similar in migraineurs and controls, the present findings contradict several previous LEP studies. Pain-evoked cerebral responses are normal and show normal habituation in migraine.

**Electronic supplementary material:**

The online version of this article (10.1186/s10194-017-0810-6) contains supplementary material, which is available to authorized users.

## Background

Migraine is a cyclic disorder as evidenced by subjective symptoms and imaging and neurophysiological studies [1–7]. Therefore, it is preferable to investigate migraine physiology repeatedly during the different phases, i.e., between, before, during and after attacks (interictal, preictal, ictal and postictal phase, respectively) [8, 9].

Laser-evoked potentials (LEP) are well suited to study the cortical response to noxious input since brief laser pulses mainly evoke cortical responses with a latency corresponding to the conduction velocity of Aδ fibers [10–14]. Aδ fiber activation yields a middle-latency component over the contralateral temporal lobe (N1) and a late biphasic vertex response (N2P2). The operculo-insular cortex and possibly the primary somatosensory cortex largely contributes to N1 [11, 15, 16], while the anterior cingulate cortex contributes to N2P2 [11]. Hence, LEP may reflect both pain-specific activation of the primary sensory cortex and cognitive and inhibitory “top-down control” aspects of pain physiology in migraine.

LEPs in migraineurs have mainly been studied by an Italian collaboration [17–24]. The results are not entirely coherent, but deficient N2P2-habituation has been observed in the interictal phase [17–19, 21, 22], a deficit that seems to persist during attacks [21]. Deficient LEP-habituation has also been observed in painful radiculopathy [25], fibromyalgia [26] and cardiac syndrome X [27]. The apparently reduced LEP-habituation in migraineurs do not differ systematically between stimulation sites [17, 18, 20–22], and whether the N1 or N2P2-potential are best suited to demonstrate an alteration is not clear [17, 20]. Accordingly, these results for LEP N2P2 should be independently confirmed [28]. N1-habituation should also be studied further in migraine as only two studies have recorded this early LEP-component [17, 20].

LEPs or other pain evoked potentials have, as far as we know, not been investigated previously in the preictal or postictal phases. In the interictal phase, lack of habituation of the nociceptive blink reflex and pain scores to repeated noxious stimuli has been shown [29, 30], with a tendency towards normalization during the migraine attack [30, 31]. However, several studies have measured evoked responses to repeated non-nociceptive stimuli in migraineurs. The results are conflicting regarding visual evoked potentials (VEP) as some studies show reduced habituation in migraineurs between attacks while others do not [9, 32, 33]. Most migraine-studies of evoked potentials habituation have focused on the interictal phase, but some have also investigated cyclic changes. One such study showed normal habituation of the standard blink reflex interictally and decreased habituation in the preictal phase [34]. However, several studies have shown an opposite effect with deficient habituation of VEP, visual evoked magnetic fields, somatosensory evoked potentials, and contingent negative variation between attacks that normalizes right before or during the attack [35–44]. One study has shown increasing loss of habituation of VEP during the interictal interval with a normalization within the migraine attack [45], while other longitudinal studies did not find VEP or brainstem auditory-evoked potential habituation differences related to the migraine cycle [4, 5]. It is accordingly of interest to extend the knowledge about general phase-related neurophysiological changes in migraine to the cortical pain-processing network.

The primary aim of the present blinded longitudinal study was to investigate generalized “third order neuron” pain network excitability in migraineurs by LEP amplitude and habituation during different stages of the migraine cycle. We examined 50 migraineurs four times to investigate intraindividual changes at both the interictal, preictal, ictal and postictal phases. We test the main working hypothesis that LEP amplitude and habituation, and subjective pain scores to laser stimulation, differs between phases. The secondary aims were to confirm previously reported deficient LEP habituation in migraineurs in the interictal phase compared to controls, and to test the effect of aura, headache laterality, years lived with migraine, and subjective pain scores on habituation and habituation-differences between phases.

## Methods

We measured LEPs and pain scores once a week for four weeks in migraineurs (mean ± SD: 6.7 ± 1.9 days between sessions) in the second half of 2012. The four sessions in one migraineur were at the same time each day for almost all subjects, but for a few subjects, it was necessary to reschedule one or two sessions. Mean variation between the latest and the earliest of the four sessions were 23 ± 28 min, and the variation was no more than an hour in 41 of 49 subjects. At most, one subject had to postpone two sessions by 3.5 h. The migraineurs completed a headache diary for four weeks before, during and four weeks after the examinations to determine how the examinations were related to the migraine attacks (i.e., interictal, preictal, ictal or postictal). We measured LEPs and pain scores once in headache-free controls. Investigators were blinded to diagnosis on subjects’ first visit and migraine phase on the subsequent visits. Co-workers performed the inclusion and follow-up, and participating subjects were specifically told not to reveal their diagnosis to the investigators.

### Subjects

Seventy-four migraineurs and 40 controls responded to an advertisement in the local newspaper, on the local hospital’s web page [46] and the Intranet within our university [47]. We screened both groups over telephone and migraineurs were evaluated by neurologists per the ICHD-II criteria for migraine with or without aura [48]. Controls could not have a headache more than once a month. If they occasionally had a headache, we asked if they had consulted a physician regarding the headache, if they experienced the headache as painful and if they used abortive medication for their headache. We excluded controls if they confirmed more than one of these three questions. Included migraineurs had an attack frequency between two and six per month and had no more than ten days with migraine attacks per month. They could use symptomatic, but not prophylactic migraine treatment. Exclusion criteria were: coexisting tension-type headache seven days or more per week in migraineurs, neurological or psychiatric diseases, sleep disorders, active infectious diseases, connective tissue diseases, metabolic, endocrine or neuromuscular diseases, other clinically relevant painful conditions including recent injuries, malignancy, previous craniotomy or cervical spine surgery, heart disease, cardiopulmonary or cerebrovascular diseases, pregnancy, medication for acute or chronic pain, antipsychotics, antidepressants, anticonvulsants or other drugs that may influence neuronal, vascular or muscular function, substance abuse, ferromagnetic implants and prophylactic allergy treatment.

Fifty migraineurs and 31 controls participated in the study. One migraineur withdrew consent after the first examination and was not included in the analysis. Three migraineurs attended only once, twice and three out of four times respectively. We excluded one control because we were unable to obtain reliable LEPs as most trials were rejected. Thus, 49 migraineurs completed a total of 190 examinations, and 30 controls completed one examination each. Table 1 shows demographic and clinical data. We report details of exclusions and dropouts in Additional file 1: Table S1.

**Table 1:** Demographic and clinical data after exclusions

	Migraineurs (*n* = 49)	Controls (*n* = 30)
Age	40 ± 10 [19–62]	38 ± 11 [21–59]
BMI	26 ± 3	25 ± 3
Women	*41* (84%)	*25* (83%)
Days since 1st day of last menstruation	17 ± 12	19 ± 10
MwoA, MA + MwoA, MA	*27* (55%), *18* (37%), *4* (8%)	NA
Years with headache	21 ± 9 [1–40]	NA
Migraine days/month^a^	1:*14*, 2:*30*, 3:*5*, 4:*0*	NA
Migraine intensity^b^	1:*2*, 2:*20*, 3:*27*, 4:*0*	NA
Headache duration^c^	16 ± 21 [0.5–72]	NA
Energy level (J) used in LEP test	4.3 ± 0.5	4.4 ± 0.4
Thresholds (J) for pinprick pain	3.7 ± 0.6	3.7 ± 0.7

^a^Migraine days/month: 0: < 1/month, 1: 1–3/month, 2: 4–7/month, 3: 8–14/month, 4: > 14/month

^b^Migraine intensity: 1: Mild, 2: Moderate, 3: Severe, 4: Extreme

^c^Average duration (hours) of an attack with or without use of symptomatic medication

Data displayed as mean ± SD [range] or *n* (%). MwoA: migraine without aura. MA + MwoA: some attacks with and some without aura (both diagnoses according to ICHD-3 Beta [88]. MA: migraine with aura (in 100% of attacks). NA: not applicable

The Regional Committees for Medical and Health Research Ethics approved the protocol, and all subjects gave their written informed consent. Migraineurs and controls received an equivalent of $ 125 and $ 30 respectively to cover expenses.

### Procedure

Painful heat stimuli were generated by a pulsed solid-state (Nd:YAP) laser (STIMUL 1340, DEKA M.E.L.A. SRL, Calenzano (FI), Italy) with a wavelength of 1340 nm. The laser stimulator settings were the same as in a previous study at our lab [49]: The pulse duration was 6 ms, a relatively short stimulus duration to maximize the N1-amplitude [50]. We set the laser beam diameter to 8 mm (area ≈ 50 mm^2^) with an energy ranging from 2 to 6.5 J (4.0–12.9 J/cm^2^). The diameter and durations are comparable to other researchers using the same type of laser [51, 52]. A diode laser aiming beam visualized the stimulation site. We recorded LEPs with a Viking Select system (Nicolet Biomedical Inc., Madison, WI, USA). The recording silver disc electrodes were placed at the Fz, Cz, Pz, T3, T4, A1 and A2 sites of the 10–20 system. The impedance was kept below 5 kΩ. The two most important analysis channels, Cz referred to the nose, and T3 referred to Fz, were preselected as recommended by the international IFCN-guidelines [53]. We used the other channels as back up to account for interindividual variation in field topography and to improve detectability of waves. For control of artifacts, we monitored the electrooculogram from a left infraorbital electrode referred to T4. The onset of stimuli triggered the recording system. The sampling rate was 1000 Hz, the sweep time was 750 ms and the filter setting was 0.2–100 Hz. Rejection level was set to ±225 μV, and total rejection rate after exclusions was 3 %. We applied online averaging [49, 54] since rejection effectively canceled artifacts and eye movements also were included in a separate channel.

Subjects lay comfortably on an examination table with laser safety glasses and acoustic earmuffs to avoid any acoustical interference at the time of stimulation [53, 55]. We delivered laser stimuli to the dorsum of the right hand between the carpal bones, metacarpophalangeal joints and second and fourth metacarpal bone. The laser beam was moved randomly within this area to avoid skin lesions and nociceptor fatigue or sensitization [56]. We measured skin temperature before the test. Because we previously observed that the recommend fixed intensity (equal to twice the mean pin-prick threshold [12]) did not always elicit pain and LEP in every healthy subject [49], we used stimulus intensities based on *intraindividual* thresholds [51, 57, 58]. First, the individual thresholds for pinprick pain were identified, starting at 2 J and increasing with 0.5 J steps [49]. The subject had to differentiate between burning pain and pinprick pain. Subjects scored pinprick pain on a verbal, numerical rating scale (NRS) with range 0 = “no pain” to 10 = “unbearable pain.” We measured the threshold twice, and we defined the pinprick threshold as the lowest intensity inducing pain in at least one of the two trials at that intensity. A tolerable intensity was 4.5 J (9.0 J/cm^2^), in most subjects, corresponding to about two times the pinprick threshold. However, 4.5 J generated too much pain in 22 subjects, and too little pain in five subjects and the energy had to be adjusted up or down (range: 3–5.5 J ≈ 6.0–10.9 J/cm^2^). The chosen intensity did generally elicit reliable N2P2 potentials [59]. We recorded two blocks of 21 stimulations with six to ten seconds between each stimulation since six seconds was recommended as the minimum interval to avoid peripheral nociceptor habituation [53]. The between-block interval was also short, between 6 and 10 s, to prevent recovery of central habituation. Subjects kept their eyes open and rated perceived pain verbally (NRS 0–10) after each stimulation to prevent LEP-amplitude decrease by distraction and drowsiness [12, 60, 61]. We stored pain scores for analysis. We used identical energy levels for all sessions within subjects. However, we did not tell participants that the energy was constant.

### Data analysis and statistics

Examinations were classified by the headache diary as interictal (more than one day before attack onset or one day after the attack ended), preictal (less than one day before attack onset), ictal (a migraine headache during the examination) and postictal (less than one day after the attack ended). We applied this definition in previous studies of pain physiology related to migraine phase [6, 62]. Eleven of the 190 examinations were unclassifiable and excluded from data analysis, mainly because they had migraines both the day before and the day after examination.

**Fig. 1:**
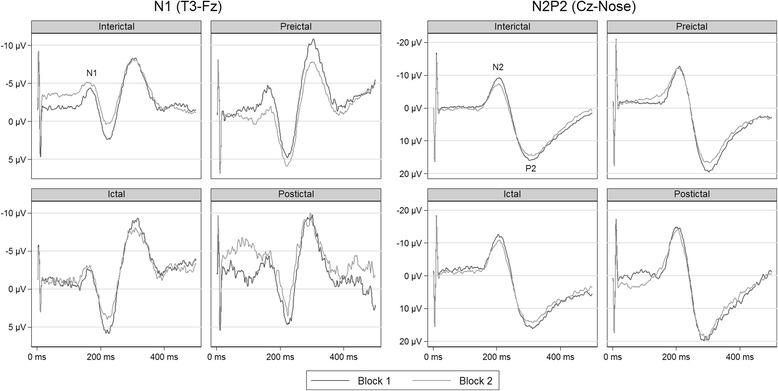
Grand average of the LEP-traces by phase. Habituation was present in all phases at both LEP-components but ictal and postictal N1, and postictal N2P2. The amplitudes in the figures are smaller than those presented in Table 2 due to slightly different LEP-latencies between participants

**Fig. 2:**
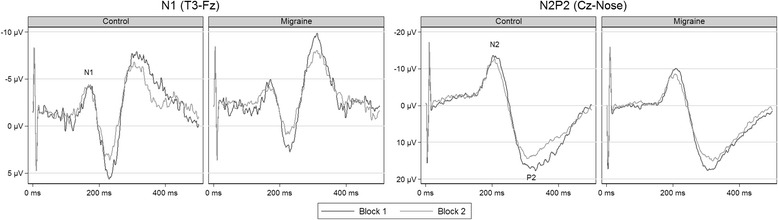
Grand average of the LEP-traces in first session interictal recordings and controls. N2P2-habituation was present in both groups, but we found no significant N1-habituation. The amplitudes in the figures are smaller than those presented in Table 4 due to slightly different LEP-latencies between participants

We analyzed in LabChart® (Version 7 pro, ADInstruments, Dunedin, New Zealand). A random number identified each LEP session and we randomized the order of the two blocks within each session. Thus, the investigator who analyzed the LEPs was blinded to diagnosis, migraine phase and order of the two blocks. The N1 and the N2P2 components were assessed, N1 at the contralateral temporal electrode (T3) against Fz (best bipolar derivation to show N1 [63]) and N2P2 at Cz against nose [53]. We measured the N1-amplitude from baseline (start of N1) to the N1 peak and the N2P2-amplitude from the most negative to the most positive peak. We had to discard some LEPs due to unrecognizable responses, too much noise/artifacts or latencies far from normal values [53]. The N1-amplitude may have a low signal-to-noise ratio, and it was not detectable in 15% of LEPs in migraineurs and 22% of LEPs in controls. These responses were included in the analysis as interval censored responses [64, 65] by setting the lower bound to zero and the upper bound to the maximal negative noise peak within the N1-time window. The exact N1-amplitude was then unknown, but we presumed that it was between zero and the largest noise peak, and we included the amplitude as an interval, a rough estimate, instead of a point estimate. We discarded recordings with technical errors, 17 of 358 in total in migraineurs. We present the grand average of all recordings by phase (Fig. 1) and by group (Fig. 2).

We analyzed data with STATA version 13.1 (StataCorp LP). We applied separate multilevel models [66] for the response variables N1, N2P2 and pain scores. The first set compared the within-subject change in migraineurs and the second set compared interictal recordings from the first exam and controls. The interaction effects were the main outcomes. For the first set, interactions-of-interest reflect differences between phases. For the second set, interactions-of-interest reflect differences between migraine and healthy subjects. We have included full model specifications in the Additional file 1.

We added explorative post hoc contrasts that tested if the slopes (i.e., habituation) at each phase, and in all phases combined, were different from zero. Also, based on the main results, we performed data-driven explorative post hoc contrasts comparing the first-block amplitudes and habituation slope of N1 between the preictal and ictal phases. We used diagnosis as a fixed factor to compare migraineurs in the interictal phase and controls. Only interictal recordings from the first exam were compared to controls because the investigator was blinded to diagnosis only on the first session, and to avoid possible long-term habituation/sensitization effects in subsequent exams. N1 and N2P2 in both sets were square rooted to improve normality.

We analyzed phase and group differences in pain scores with pain scores from both blocks combined consecutively into one continuous time variable which was interacted with phase or group, respectively. The time variable was centered at its mean and divided by 10. Thus, the regression constant shows the average pain score and the habituation coefficient the change in pain score per ten stimulations. We tested group differences in pain thresholds and laser intensity with independent samples Student’s t-test and present results as 95% confidence intervals (CI). Thus we consider intervals not containing zero to be significant at a level of *p* < 0.05. We have back transformed the data and tabulate result-parameters in the original scale. However, results are presented as transformed units in the Additional file 1.

We extended the original models to test the effect of additional variables. We specified four separate models that estimated the phase-differences in habituation for 1) migraineurs with and without aura, 2) sessions differentiated by headache laterality, 3) by years lived with migraine, and 4) by pain scores. Headache laterality was classified by the related attack if the phase was preictal, ictal or postictal. Interictal recordings were classified by the side the subject most commonly experienced headache, either left, right or bilateral. Sixteen interictal recordings had an equal amount of left and right-sided unilateral migraine and were not included in the laterality-analysis. We included age, migraine intensity, and migraine frequency as control variables (not included in the interactions) in the extended model 3 that estimated the effect of years lived with migraine.

We conducted three additional analyses to explore the relationship between habituation and number of days to next attack. We conducted these analyses in two steps, first with interictal phase only and then with both interictal and preictal phases included. We interpret the interaction effects in the latter analyses as the interictal-preictal day-to-day change in habituation towards the next migraine attack. Also, we performed a secondary set of analyses with a three-day limit to test if postictal phase-related LEP-changes last longer than 24 h after the attack.

With 30 controls and 50 migraineurs, the statistical power to detect a low medium-sized effect equal to 0.65 SD [67] based on a two-sample Student’s t-test is 80%. As we estimated to have approximately 20 pairs for intraindividual phase-related comparisons, power (based on paired Student’s t-tests) to detect a similar medium-sized effect (0.65 SD) was calculated to 83%.

## Results

### Analyses by phase

Table 2 shows means and standard deviations of N1 and N2P2-amplitudes, and pain scores by phase.

**Table 2:** N1 and N2P2-amplitudes, and pain scores by phase and block

			N1 (μV)		N2P2 (μV)		Pain scores	
	*N*	*n*	Block 1	Block 2	Block 1	Block 2	Block 1	Block 2
Interictal	44	99	6.6 (3.5)	5.9 (2.6)	40.2 (16.6)	35.2 (13.8)	4.2 (1.9)	4.3 (2.0)
Preictal	26	36	7.9 (5.0)	6.3 (3.6)	42.3 (13.4)	37.3 (11.2)	4.1 (1.9)	4.4 (2.0)
Ictal	19	21	5.7 (2.2)	5.0 (2.2)	39.6 (12.8)	34.6 (10.1)	4.4 (1.7)	4.7 (1.9)
Postictal	13	15	6.8 (3.0)	4.9 (3.7)	45.7 (13.7)	43.2 (12.5)	5.4 (2.6)	5.7 (2.7)

Mean (SD) N1 and N2P2-amplitudes, and pain scores. The means were calculated in two steps; first, phase-specific means for each subject (most subjects had two or more measurements classified within the same phase), before phase-specific means in all subjects combined. Because some N1-amplitudes were interval censored, i.e., defined only by a minimum and maximum with the actual value somewhere in between, the interval midpoints were used as approximate estimates to calculate the means. *N:* number of subjects with at least one recording at the respective phase. *n*: total number of recordings at the respective phase

**Fig. 3:**
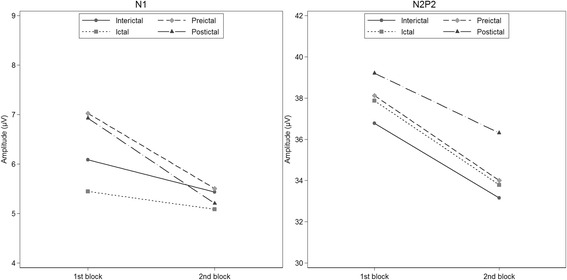
Estimates of N1 (left) and N2P2-amplitude (right) habituation by phase

N1-habituation was significant in the interictal phase as shown by the negative coefficient of block (Fig. 3 and Table 3). The degree of habituation was not different between the interictal phase and the preictal, ictal and postictal phases respectively (interaction effects in Table 3). However, post hoc contrasts showed significant habituation in the preictal phase (95% CI [−2.71, −0.33] μV/block), but not in the ictal and postictal phases (95% CI [−1.29, 0.57] and [−3.44, 0.00] μV/block, respectively). The habituation in all phases combined was significant (95% CI [−1.40, −0.38] μV/block). The contrast of the difference in habituation between the preictal and ictal phase was not significant (95% CI [−0.54, 2.86] μV/block). Neither the first-block amplitudes nor the combined first and second-block amplitudes differed between phases, but the post hoc contrast that compared first-block amplitudes between the preictal and ictal phases showed a tendency towards lower first-block amplitudes in the ictal phase (95% CI [−3.20, 0.04] μV).

**Table 3:** Estimated magnitudes and habituation of N1, N2P2 and pain scores by phase

	N1 (μV)		N2P2 (μV)		Pain scores	
	Coef.	95% CI	Coef.	95% CI	Coef.	95% CI
Main effects						
Preictal	0.939	[−0.476, 2.353]	1.342	[−1.993, 4.677]	−0.01	[−0.36, 0.34]
Ictal	−0.641	[−1.753, 0.470]	1.088	[−2.862, 5.039]	0.23	[−0.28, 0.74]
Postictal	0.839	[−0.487, 2.166]	2.426	[−2.419, 7.272]	0.37	[−0.35, 1.10]
Habituation	−0.653*	[−1.315, −0.001]	−3.623***	[−5.147, −2.098]	0.21***	[0.09, 0.33]
Interaction effects						
Preictal × Habituation	−0.868	[−2.283, 0.547]	−0.497	[−3.445, 2.451]	0.07	[−0.10, 0.24]
Ictal × Habituation	0.293	[−0.746, 1.331]	−0.455	[−4.086, 3.176]	0.06	[−0.18, 0.31]
Postictal × Habituation	−1.070	[−2.890, 0.749]	0.726	[−3.657, 5.108]	0.12	[−0.17, 0.41]
Constant	6.088	[5.186, 6.989]	36.783	[33.002, 40.565]	4.11	[3.56, 4.67]

The constant represents interictal first-block or mean pain score responses, the first three main effects are first-block amplitude or pain score differences from the interictal phase and the fourth “Habituation” main effect is the difference between first and second block, or the linear change of pain scores, in the interictal phase. The interaction effects represent habituation differences between the interictal phase and the preictal, ictal and postictal phases, respectively. Thus, the significant coefficients are interpreted as decreased second-block N1 and N2P2-amplitudes, and linear increase in pain scores, in the interictal phase, i.e. interictal N1 and N2P2 habituation and subjective pain sensitization. Lack of significant interaction effects are interpreted as no habituation differences between the interictal phase and the other phases. Random effects estimates are shown in Supplementary Table 2. * *p* < 0.05, ** *p* < 0.01, *** *p* < 0.001

The N2P2-amplitude change from the first to the second block was significant in the interictal phase, and none of the interactions were significant (Fig. 3 and Table 3), interpreted as interictal habituation with no differences between phases. Post-hoc contrasts showed significant habituation in both the preictal and ictal phases (95% CI [−6.66, −1.58] and [−7.38, −0.77] μV/block, respectively), but not in the postictal phase (95% CI [−7.01, 1.22] μV/block). The habituation in all phases combined was significant (95% CI [−4.90, −2.55] μV/block). N2P2-amplitude sizes did not differ between phases.

Pain scores increased linearly in the interictal phase (95% CI [0.11, 0.33] NRS-change/10 stimuli, Table 3). The linear increase, i.e., sensitization of pain scores, was not different between phases. Mean pain scores did not differ between phases.

We present complete results from the secondary analyses in the Additional file 1. N1 and N2P2 first-block amplitudes and habituation did not differ between migraineurs with and without aura as none of the three-way or two-way interactions were significant. Amplitudes and habituation did not differ between a left and right-sided migraine. Subjects with a bilateral migraine had reduced N2P2-habituation (more positive slope) in the postictal phase compared to the interictal phase (95% CI [0.07, 25.3] μV/block), and the same tendency was present in the ictal phase (95% CI [−0.01, 19.8] μV/block). The more years lived with migraine; the less was the N2P2-habituation in the preictal phase compared to the interictal phase (95% CI [0.11, 0.82] μV/block/year adjusted for age). No other interaction effects were significant.

Both N1 and N2P2 interictal first-block amplitudes correlated with pain scores (95% CI [0.08, 0.93] and [1.74, 3.65] μV/unit pain score for N1 and N2P2, respectively). The interactions between phase and pain score were not significant, that is, the correlations were not different between phases. Habituation of N1 and N2P2-amplitudes did not correlate with a change in pain scores from the first to the second block.

The analyses that explored the relationship between habituation and number of days to next attack showed no significant interactions. Thus, there was no interictal day-to-day linear change in habituation towards the next migraine attack. Changing the definition of the postictal phase from a one-day limit to a three-day limit did not alter the interpretation of LEP-habituation. Habituation of pain scores did not change by changing the definitions of the phases, although the mean pain score was significantly increased in the postictal compared to the interictal phase (95% CI [0.11, 1.28] unit pain score).

### Analyses by diagnosis

Table 4 shows means and standard deviations of N1 and N2P2-amplitudes, and pain scores by group.

**Table 4:** N1 and N2P2-amplitudes, and pain scores in migraineurs in the interictal phase and controls

		N1 (μV)		N2P2 (μV)		Pain scores	
	*N*	Block 1	Block 2	Block 1	Block 2	Block 1	Block 2
Migraineur	29	7.0 (4.0)	6.1 (3.1)	38.7 (17.5)	33.5 (14.5)	4.2 (2.0)	4.1 (2.3)
Control	30	8.5 (8.6)	7.8 (7.5)	41.1 (16.9)	35.2 (15.3)	3.6 (1.6)	3.5 (1.6)

Mean (SD) N1 and N2P2-amplitudes, and pain scores. The migraine group consists of interictal recordings from the first session. The means and SD of N1-amplitudes are calculated with the interval midpoints of interval censored responses. *N:* number of subjects with a recording of at least one block

**Fig. 4:**
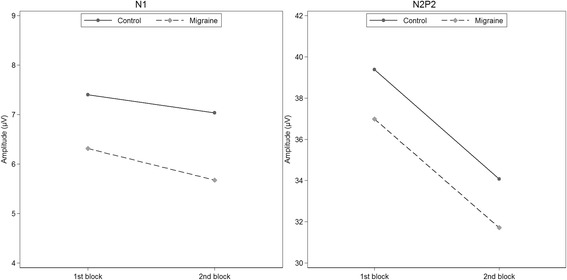
Estimates of N1 (left) and N2P2-amplitude (right) habituation in interictal recordings and controls

Controls did not show habituation of the N1 amplitude, and habituation did not differ between migraineurs and controls (Fig. 4 and Table 5). Post-hoc contrasts showed no habituation in the groups combined (95% CI [−1.32, 0.30] μV) and no habituation in the migraine group (95% CI [−1.77, 0.49] μV). Neither the first-block amplitudes nor the combined first and second-block amplitudes differed between migraineurs and controls.

**Table 5:** Estimated magnitudes and habituation in migraineurs in the interictal phase and controls

	N1 (μV)		N2P2 (μV)		Pain scores	
	Coef.	95% CI	Coef.	95% CI	Coef.	95% CI
Main effects						
Migraine	−1.014	[−3.767, 1.739]	−2.395	[−10.707, 5.917]	0.60	[−0.32, 1.52]
Habituation	−0.374	[−1.527, 0.778]	−5.307***	[−9.232, −1.383]	0.10	[−0.06, 0.26]
Interaction effect						
Migraine × Habituation	−0.268	[−1.875, 1.339]	−0.036	[−4.781, 4.854]	0.13	[−0.09, 0.35]
Constant	7.365	[5.070, 9.660]	39.382	[33.531, 45.234]	3.54	[3.01, 4.07]

The constant represents first-block amplitude or pain score responses in controls. The main effect of migraine represents the first-block amplitude or pain score difference between groups. The main effect of habituation represents the difference between first and second block amplitudes, or linear change in pain scores, in the control group. The interaction effect represents the habituation-difference between groups. Thus, the significant coefficient is interpreted as N2P2 habituation in the control group. The corresponding interaction effect is not significant, indicating no difference in habituation between controls and migraineurs in the interictal phase. Random effects estimates are shown in Supplementary Table 3. * *p* < 0.05, ** *p* < 0.01, *** *p* < 0.001

The N2P2-amplitude decreased from the first to the second block in controls, and the decrease was not different between controls and migraineurs (Fig. 4 and Table 5). Post-hoc contrasts confirmed a significant habituation in migraineurs (95% CI [−8.07, −2.48] μV). Overall amplitudes were not different between groups (95% CI [−9.87, 5.11] μV).

The linear change in pain scores was not significantly different from zero in controls (95% CI [−0.06, 0.27] NRS-change/10 stimuli) and did not differ between migraineurs and controls (95% CI [−0.09, 0.35] NRS-change/10 stimuli, Table 5). Pain thresholds and stimulation intensities were not different between groups (Student’s t-test 95% CI [−0.37, 0.34] and [−0.04, 0.44], respectively).

## Discussion

As far as we know, this is the first study to measure cyclic changes of LEP N1-habituation in migraine. Our results show habituation of both N1 and N2P2 amplitudes in all phases combined. In line with the overall responses, both interictal and preictal N1 and N2P2 habituated. Habituation of N2P2 was present in the ictal phase as well. The deficient ictal habituation of N1 was only present in the post hoc contrasts, not in the main analysis, and the number of ictal recordings was relatively small (*n* = 21). Thus, we interpret the finding of deficient ictal N1 habituation with caution, and we believe that it needs to be replicated.

It has been suggested that lack of habituation and normal or slightly decreased first-block amplitudes are functional properties of migraine between attacks [33]. These properties seem to normalize during the attack, at least for non-noxious evoked potentials [9]. The reduced habituation may be due to thalamocortical “dysrhythmia” [9], as suggested by both high-frequency [68] and low-frequency oscillations [69]. This proposed dysrhythmia may reduce thalamic control of the sensory cortices and render the pre-activation level low [33]. Thalamocortical dysrhythmia has been suggested in several diseases, e.g., tinnitus [70], neuropsychiatric disorders [71, 72] and chronic pain [73, 74]. However, in the present study, we found normal interictal LEP-habituation, although we observed deficient habituation and a tendency towards lower first-block amplitude of N1 during attacks, i.e., no tendency towards “normalization.” Our present findings do not support the concept of a generally reduced interictal habituation in migraine.

On the other hand, the discrepancy between a preserved ictal N2P2-habituation, as opposed to a subtle deficient N1-habituation, suggests a centrally mediated ictal alteration [75]. The N1-component likely reflects the sensory-discriminative component of pain whereas the N2P2-component reflects the motivational and cognitive component of pain [59]. Thus, migraine pain seems to primarily affect sensory processes rather than cognitive, in contrast to the effects of sleep deprivation shown in one study [76].

In the present study, we could not reproduce altered N2P2-habituation or amplitude during attacks. This result contradicts the findings of other smaller studies. One study has shown reduced hand and face N2P2-habituation in interictal recordings (*n* = 14) compared to controls (*n* = 10) and a similar habituation deficit during attacks (*n* = 8) [21]. Two studies (*n* = 10 and 18) have demonstrated an increased N2P2-amplitude during compared to between attacks [23, 24]. Two of the studies included subjects with mean migraine frequency close to chronic migraine (we had none), and this could have contributed to the discrepancy between their and our results [21, 24].

The post hoc contrasts showing a lack of habituation of both N1 and N2P2-amplitudes in the postictal phase should be interpreted with caution as the number of postictal measurements were lower than for the other phases. Accordingly, this negative finding may be a result of rather low statistical power. Importantly, the main analyses showed no significant differences between habituation slopes, and Fig. 3 indicates that habituation is present in the postictal phase as well.

Pain scores increased linearly throughout the stimulation, in contrast to the decrease in N1 and N2P2-amplitudes. However, the negative correlations between pain score and amplitudes were not significant. Mean pain scores and linear change of pain scores were not different between phases. Previous studies have shown reduced laser-pain thresholds during the attack [23, 24], and one study has shown increased pain scores during compared to between attacks by stimulation on both sides of the face but not the hands [21].

Habituation did not differ between migraineurs with and without aura. This finding cannot be compared to previous studies of LEP-habituation as they only included migraineurs without aura [17–22]. The positive correlation between pain scores and LEP amplitudes fits with earlier migraine studies [18–21, 24]. Interestingly, subjects with a bilateral headache had deficient postictal habituation compared to lateralized headache. We speculate if bilaterality represents excessive headache load, but a similar habituation deficiency was not observed for the load-parameter “years lived with migraine” (controlled for age, intensity, and frequency). However, *preictal* habituation was less pronounced in subjects with more migraine-years. Thus, there is some evidence of subtle changes of habituation by clinical features in proximity to attack, but the subgroups are small (e.g., only ten interictal and seven postictal sessions were associated with a bilateral headache), and the analyses many, hence it may be a random type 1 error.

Migraineurs in the interictal phase and controls showed no group differences. The amplitudes and pain scores were similar, and both groups had no significant N1-habituation but significant N2P2-habituation, and no linear change in pain scores. These findings are in contrast with some of the previously published results. The group differences in amplitude have varied considerably between studies. Group differences in N1 or N2P2-amplitudes after hand or face stimulation have only been reported in small studies (*n* = 9–14 in each group) [18–20]. There were no amplitude differences between groups in a larger study (*n* = 24 and 28) [17], including the present study (*n* = 29 and 30). In contrast to our results, one small study has reported habituation of N1-amplitudes in controls after hand stimulation compared to no habituation in migraineurs [20]. The same study showed no habituation in controls after face stimulation, but an extreme amplitude *potentiation* of more than 90% in migraineurs [20]. Valeriani et al. [17] showed N1-habituation after hand stimulation in controls (but not in migraineurs) and no habituation in either group after face stimulation. However, it is unclear if the migraine group had significantly reduced amplitude habituation compared to the control group because the authors did not compare the degree of habituation between groups statistically.

N2P2-habituation was reduced in migraineurs compared to controls after face [17–19, 21, 22] and hand [17, 18, 21, 22] stimulation in most previous studies, although one study showed no differences [20]. The reliability of significant effects in small studies is low even in the absence of other biases [77]. Independent replications are thus necessary to increase the reliability of the estimated effects. Based on the results of the present larger and blinded study, it seems reasonable to conclude that habituation and amplitudes after hand stimulation are not different in migraineurs compared to non-headache control subjects, or if they are different, the differences are small. It has been argued that deficient habituation is a neurophysiological hallmark of migraine [78, 79]. However, as for VEP [32], the contradictory findings of LEP studies do not support that hypothesis.

Pain score changes by stimulus repetitions did not differ between migraineurs and controls in the present study. Previous studies have demonstrated similar findings [18, 20], although one study found differences represented by pain score habituation in controls and potentiation in migraineurs [21]. Also, de Tommaso et al. [19] demonstrated pain score habituation in controls and only a habituation tendency in migraineurs, but they did compare the groups statistically.

Variation in applied methods may explain some of the discrepancy mentioned above (Table 6). For instance, three of the previous studies recorded LEPs from three blocks with a five-minute break in between while we recorded two blocks without delay. Therefore, the less pronounced habituation shown by those studies may represent late effects only present after about ten minutes of stimulation. A similar late effect has been shown in radiculopathy patients where the habituation of N2P2 was normal in the first three blocks of 25 stimuli (inter-stimuli interval 8–12 s, no break between blocks), but deficient in the fourth [25].

**Table 6:** Studies on LEP-habituation in migraine

Author	Aim/ habituation measure		♀	♂	Age	Incl	Excl	Freq	Blinding	Laser	Intensity	Block	Stim	ISI	IBI	Comp	Sites	Main habituation results^a^
Valeriani 2003 (17)	Habituation/ % change	Mig:	14	10	33.7 ± 8.2	NR	NR	NR	NR/NR	CO_2_	2.5 × STh	3	15–30^b^	8–12	5 m	N1 N2P2	Bilateral hands and face^c^	N1 hand: CO↘ Mig→
										10.6 μm								N1 face: CO → Mig→
		CO:	17	11	32.5 ± 6.4	NR	NR			2 mm								N2P2 hand: CO↘ > Mig↘
										10 ms								N2P2 face: CO↘ Mig→
de Tommaso 2005 (19)	Habituation/ ANOVA	Mig:	9	5	22–53	NR	NR	NR	NR/NR	CO_2_	7.5 W	3	21	10	10s	N2P2	Rigth supraorbital	N2P2 face: CO↘ Mig→
										10.6 μm								
		CO:	7	3	21–50	NR	NR			2.5 mm								
										20 ms								
de Tommaso 2005 (21)	Interictal-ictal habituation/ ANOVA	Mig:	14	0	18–40	NR	NR	9.4 ± 4.6	NR/Yes	CO_2_	7.5 W	3	20	10	10s	N2P2	Bilateral hands and supraorbital	N2P2 hand: CO↘ > Mig→
										10.6 μm								
		CO:	10	0	22–36	NR	NR			2.5 mm								N2P2 face: CO↘ > Mig→
										20 ms								
de Tommaso 2009 (18)	Pre-menstrual habituation/ Ratio	Mig:	9	0	26 ± 6.8	Outp	11	4 ± 2.94	NR/Yes	CO_2_	7.5 W	3	20	10–15	5 m	N2P2	Right hand and supraorbital	N2P2 hand: CO > Mig
										10.6 μm								
		CO:	10	0	26.8 ± 5.3	Hosp staff	15			2 mm								N2P2 face: CO > Mig
										25 ms								
Di Clemente 2013 (20)	Intervention^d^ / % change	Mig:	9	4	38.5 ± 12.0	NR	9	5.8 ± 2.2	NR/Yes	Nd:YAP	2.5 × STh	3	15	10	5 m	N1 N2P2	Right hand and supraorbital	N1 hand: CO > Mig
										NR								N1 face: CO > Mig
		CO:	10	5	30.9 ± 5.7	NR	0			NR								N2P2 hand: CO = Mig
										NR								N2P2 face: CO = Mig
Vecchio 2016 (22)	Intervention^e^/ % change	Mig:	23	9	35.5 ± 10	NR	1	6 ± 2.5	NR/Yes	CO_2_	PTh + 1 W	3	7	10	10s	N2P2	Right hand and supraorbital	N2P2 hand: CO > Mig
										10.6 μm								
		CO:	12	4	36.0 ± 11	NR	0			2 mm								N2P2 face: CO > Mig
										25 ms								
Uglem 2017	Habituation by phase/ Multilevel mixed mod.	Mig:	41	8	40 ± 10	Adv	Yes^f^	4^g^	Yes/Yes	Nd:YAP	3–5.5 J	2	21	6–10	10s	N1 N2P2	Right hand	N1 hand: CO → =Mig→
										1.34 μm								
		CO:	25	5	38 ± 11	Adv				8 mm								N2P2 hand: CO↘ = Mig↘
										6 ms								

♀: number of females. ♂: number of males. Age is presented in mean ± SD or range. Incl: inclution/method of recruitment. Excl: number of exclusions. Freq: migraine attack frequency in mean ± SD days/monts. Blinding: blinding of investigators during examination/blinding of investigators during LEP-analysis. Laser: Type of laser, wavelength, beam diameter and stimulus duration. Block: number of blocks. Stim: number of stimuli per block. ISI: interstimuli interval in seconds. IBI: interblock interval. Comp: analyzed LEP-components. Mig: migraine. CO: control. NR: not reported. Outp: outpatients. Hosp staff: hospital staff. Adv: recruited by advertisement. STh: sensory threshold. PTh: pain threshold. ↘: habituation present. →: no significant habituation. >: increased habituation in the control group compared to the migraineur group. =: no significant difference in habituation between the groups

^a^Only results regarding habituation of N1 and/or N2P2-amplitudes are shown. The studies differ in how they analyzed habituation as some reported habituation in each group separately (results indicated by ↘ or →), some reported comparisons between groups (results indicated by > or =), and some reported both. Valeriani 2003 (17) reported both results for N2P2 hand, but not for the other amplitudes.

^b^Three blocks with 15 stimuli at each side of the face and three blocks with 30 stimuli at each hand.

^c^Data were collected from three centers that stimulated two different face regions. The perioral region was stimulated in all migraineurs and 17 of the control subjects, while the supraorbital region was stimulated in 13 controls. The reported number of controls from each center does not add up to the reported total of 28 controls.

^d^The study examined the effect of topiramate on LEPs.

^e^The study investigated the effect of transcranial direct current stimulation of the left primary motor cortex and left dorsolateral prefrontal cortex on LEPs.

^f^Exclutions and dropouts are presented in Additional file 1: Table S1.

^g^Frequency was recorded in blocks. The three blocks 1–3, 4–7 and 8–14 days/month contained 14, 30 and 5 migraineurs, respectively.

### Strengths and limitations

The major strengths of this study are its relatively large size with a longitudinal design and rigorous blinding both during data collection and analysis. The level of arousal, attention, and distraction may affect LEPs [60, 61]. Therefore, within-study consistency of the laser stimulation procedure is important. Especially when comparing groups, blinding of the investigators performing the stimulations becomes a necessity. Unfortunately, none of the previous migraine LEP-studies reported blinding of the investigators during stimulation, although the majority analyzed the LEPs blind to diagnosis (Table 6).

The solid-state laser used in this study differs from the CO_2_-lasers employed in previous studies in that it produces a laser beam with shorter wavelength with deeper skin penetration that activates nociceptors more directly. This increases the amplitude of N1 and N2 and shortens the latency of N2P2, but the distribution of brain generators remains equal [80]. We do not believe that these differences, or the subtle differences in target-intensity, can explain the discrepancy between previous and present results.

The longitudinal design ensured a substantial number of interictal and preictal measurements and an acceptable number of ictal measurements. The postictal estimates are the least reliable due to the lowest number of measurements in that phase [77], although the number is comparable to previous migraine LEP-studies [21, 23, 24]. Only first-session responses were included in the migraineur versus control analyses because the investigator could not be blinded to diagnosis for the subsequent sessions, and to avoid possible long-term habituation/sensitization effects in later exams. Nevertheless, the number of interictal responses in this study was equal to [17, 22] or considerably larger than in the previous studies whose findings we attempted to reproduce [18–21].

We always stimulated the right hand regardless of the side the migraineur predominantly experienced headache. We found no habituation differences of LEPs obtained ipsilateral and contralateral to a migraine headache in accordance with previous findings [17]. Hence, it is seemingly not necessary to adjust the stimulated side according to headache laterality. We did not collect information on clinical allodynia, which could be of importance as an explanatory variable. We recruited both migraineurs and controls from the general population, and this design may enhance the generalizability of our results to the standard migraine population [81]. Having a first-degree relative that suffers from migraine may influence the habituation in controls [82]. However, we found habituation in both groups, not lack of habituation, which would be the expected finding if migraine-related genes biased our control group. Also, only four of the controls in our study had a positive family history of migraine, and excluding them from the analyses did not change the conclusions (results not reported).

The use of symptomatic treatment may have influenced the results as both triptans and non-steroidal anti-inflammatory drugs may reduce the amplitudes during the ictal phase [83]. It is unlikely that the medication influenced other phases than the ictal phase due to short half-life. Lack of facial stimulation can also be considered a limitation. However, hand and face LEP habituation seems to agree quite well in previous studies (Table 6), and this is to be expected as LEP reflects activation of a large part of the bilateral cortical pain network, and our aim was to study the generalized effects. Also, other modalities like pain thresholds have shown abnormalities in hands (and face) [84–86], suggesting an eventual thalamocortical dysfunction in migraine, in line with the development of cutaneous allodynia demonstrated by Burstein et al. [87]. Nevertheless, the present results are valid only for the more global pain function in migraine. It is necessary to do a similar study with face stimulation to conclude about lateralized second order trigeminal medullar afferent sensitization can be detected by LEP-abnormalities.

Previous studies have calculated habituation differently (Table 6). The method we chose included all available data and estimated the amplitude-change without prior calculation/manipulation of the dependent variable. Also, the approach did not use listwise deletion of cases with missing values, as would be the case with ANOVA. We were thus able to compare all four phases in one model. We also included the N1-responses where the signal to noise ratio was too low, as interval censored variables instead of discarding them, to avoid exclusion bias [65].

## Conclusion

Both imaging and neurophysiological studies have shown phasic alterations in migraineurs. However, we only found evidence of a subtle alteration of habituation of cerebral responses to painful laser stimulation in the ictal phase. We found comparable LEP-amplitudes and habituation to dorsal hand stimulation in migraineurs in the interictal phase and headache-free controls. Thus, in contrast to some previous studies, we conclude that cerebral responses to painful laser stimulation are normal interictally in migraineurs. LEPs seem to be stable throughout the migraine cycle, but we could not exclude small changes and recommend further studies on phase-related changes in pain-physiology.

## Additional file

Additional file 1: Table S1. Number of migraineurs and controls at each recruitment stage. **Table S2.** Estimated magnitudes and habituation of N1, N2P2 and pain scores by phase. **Table S3.** Estimated magnitudes and habituation in migraineurs in the interictal phase and controls. **Table S4.** Estimated amplitudes and habituation of N1 and N2P2 by phase and the effect of aura. **Table S5.** Estimated amplitudes and habituation of N1 and N2P2 by phase and the effect of headache laterality. **Table S6.** Estimated amplitudes and habituation of N1 and N2P2 by phase and the effect of years lived with migraine (YwM). **Table S7.** Estimated amplitudes and habituation of N1 and N2P2 by phase and the effect of pain scores. (DOCX 39 kb)

## Abbreviations

CI: Confidence interval
LEP: Laser-evoked potentials
NRS: Numerical rating scale
VEP: Visual evoked potentials
