# Supplementary material for: Habituation of laser-evoked potentials by migraine phase: a blinded longitudinal study
Source: J Headache Pain. 2017 Oct 2;18(1):100. doi: 10.1186/s10194-017-0810-6 (PMC5624861; doi:10.1186/s10194-017-0810-6)
Supplement: Additional file 1: Table S1. — Number of migraineurs and controls at each recruitment stage. Table S2. Estimated magnitudes and habituation of N1, N2P2 and pain scores by phase. Table S3. Estimated magnitudes and habituation in migraineurs in the interictal phase and controls. Table S4. Estimated amplitudes and habituation of N1 and N2P2 by phase and the effect of aura. Table S5. Estimated amplitudes and habituation of N1 and N2P2 by phase and the effect of headache laterality. Table S6. Estimated amplitudes and habituation of N1 and N2P2 by phase and the effect of years lived with migraine (YwM). Table S7. Estimated amplitudes and habituation of N1 and N2P2 by phase and the effect of pain scores. (DOCX 39 kb) [file 10194_2017_810_MOESM1_ESM.docx]

Additional file 1

**Table S1.** Number of migraineurs and controls at each recruitment stage.

|  | **Migraine** | | |  | **Controls** | | |
| --- | --- | --- | --- | --- | --- | --- | --- |
|  | ***N*** | **Excl.** | **Dropout** |  | ***N*** | **Excl.** | **Dropout** |
| Telephone screening | 74 | 6 | 13 |  | 40 | 2 | 7 |
| Inclusion by neurologist | 55 | 2 | 3 |  |  |  |  |
| 1st exam | 50 | 1 | 1 |  | 31 | 1 |  |
| 2nd exam | 48 |  | 1 |  |  |  |  |
| 3rd exam | 47 |  | 1 |  |  |  |  |
| 4th exam | 46 |  |  |  |  |  |  |

One migraineur withdrew consent after the first exam and was excluded. One control was excluded because we were unable to obtain reliable LEPs as most trials were rejected. Drop outs account for six missing tests in migraineurs. *N:* number of subjects. Excl.: number of excluded subjects. Dropout: number of subjects who dropped out due to personal reasons.

# Statistical details

Migraine phase and block number were dummy-coded with the interictal phase and first block as a baseline when analyzing N1 and N2P2. Phase, block, and their interactions were included as fixed factors. Thus, the constant represented the mean interictal first-block amplitudes, and the main effects represented the difference in first-block amplitudes from the interictal to the preictal, ictal, and postictal phases, and the interictal amplitude change from the first to the second block. The interaction effects represented differences in change from the first to the second block from the interictal to the preictal, ictal and postictal phases.

Dependencies in the data were accounted for by including subject and session (1,2,3 or 4) as random factors in the first set and subject as a random factor in the second set of the multilevel models. Thus, the first set was modeled as a three-level model with responses nested in sessions nested in subjects, and the second set as a two-level model with responses nested in subjects. The random slope of time was included in the analysis of pain scores. Normality of residuals and random factors were controlled with histograms. N1 and N2P2 in both sets were square rooted to improve normality. A robust variance covariance estimator was applied.

The extended models were extensions of the original models with three-way interactions between the included variables and all two-way interactions.

**Table S2.** Estimated magnitudes and habituation of N1, N2P2 and pain scores by phase.

|  | **N1 (µV^0.5^)** | |  | **N2P2 (µV^0.5^)** | |  | **Pain scores** | |
| --- | --- | --- | --- | --- | --- | --- | --- | --- |
|  | **Coef.** | **95% CI** |  | **Coef.** | **95% CI** |  | **Coef.** | **95% CI** |
| **Main effects** |  |  |  |  |  |  |  |  |
| Preictal | 0.171* | [-0.083, 0.425] |  | 0.110*** | [-0.162, 0.381] |  | -0.01*** | [-0.36, 0.34] |
| Ictal | -0.123* | [-0.339, 0.092] |  | 0.089*** | [-0.233, 0.411] |  | 0.23*** | [-0.28, 0.74] |
| Postictal | 0.153* | [-0.085, 0.391] |  | 0.197*** | [-0.192, 0.585] |  | 0.37*** | [-0.35, 1.10] |
| Habituation | -0.126* | [-0.251, -0.001] |  | -0.306*** | [-0.434, -0.178] |  | 0.21*** | [0.09, 0.33] |
| **Interaction effects** |  |  |  |  |  |  |  |  |
| Preictal × Habituation | -0.157* | [-0.416, 0.102] |  | -0.037*** | [-0.283, 0.209] |  | 0.07*** | [-0.10, 0.24] |
| Ictal × Habituation | 0.054* | [-0.149, 0.256] |  | -0.034*** | [-0.338, 0.269] |  | 0.06*** | [-0.18, 0.31] |
| Postictal × Habituation | -0.199* | [-0.547, 0.148] |  | 0.071*** | [-0.288, 0.429] |  | 0.12*** | [-0.17, 0.41] |
| **Constant** | 2.662* | [2.493, 2.832] |  | 6.065*** | [5.753, 6.377] |  | 4.11*** | [3.56, 4.67] |
| **Random effects** |  | |  |  | |  |  | |
| Level 3: subject (intercept) | 0.178 | |  | 1.014 | |  | 3.31 | |
| Level 2: session (intercept) | 0.045 | |  | 0.170 | |  | 0.86 | |
| Level 2: time (slope) |  | |  |  | |  | 0.11 | |
| Level 1: residuals | 0.156 | |  | 0.207 | |  | 1.90 | |

N1 and N2P2-amplitudes were square root transformed to improve normality of residuals and coefficient magnitudes should be interpreted accordingly. Results in the paper are presented in the original scale. The constant represents interictal first-block or mean pain score responses, the first three main effects are first-block amplitude or pain score differences from the interictal phase and the fourth “Habituation” main effect is the difference between first and second block, or the linear change of pain scores, in the interictal phase. The interaction effects represent habituation differences between the interictal phase and the preictal, ictal and postictal phases, respectively. Thus, the significant coefficients are interpreted as decreased second-block N1 and N2P2-amplitudes, and linear increase in pain scores, in the interictal phase, i.e. interictal N1 and N2P2 habituation and subjective pain sensitization. Lack of significant interaction effects are interpreted as no habituation differences between the interictal phase and the other phases. Random effect estimates are displayed as variances. * *p* < 0.05, ** *p* < 0.01, *** *p* < 0.001.

**Table S3.** Estimated magnitudes and habituation in migraineurs in the interictal phase and controls.

|  | **N1 (µV^0.5^)** | |  | **N2P2 (µV^0.5^)** | |  | **Pain scores** | |
| --- | --- | --- | --- | --- | --- | --- | --- | --- |
|  | **Coef.** | **95% CI** |  | **Coef.** | **95% CI** |  | **Coef.** | **95% CI** |
| **Main effects** |  |  |  |  |  |  |  |  |
| Migraine | -0.181 | [-0.667, 0.305] |  | -0.194*** | [-0.867, 0.479] |  | 0.60 | [-0.32, 1.52] |
| Habituation | -0.065 | [-0.264, 0.134] |  | -0.438*** | [-0.760, -0.117] |  | 0.10 | [-0.06, 0.26] |
| **Interaction effect** |  |  |  |  |  |  |  |  |
| Migraine × Habituation | -0.056 | [-0.343, 0.232] |  | -0.012*** | [-0.408, 0.384] |  | 0.13 | [-0.09, 0.35] |
| **Constant** | 2.892 | [2.496, 3.289] |  | 6.276*** | [5.809, 6.742] |  | 3.54 | [3.01, 4.07] |
| **Random effects** |  | |  |  | |  |  | |
| Level 2: subject (intercept) | 0.214 | |  | 1.339 | |  | 3.06 | |
| Level 2: time (slope) |  | |  |  | |  | 0.07 | |
| Level 1: residuals | 0.111 | |  | 0.283 | |  | 2.22 | |

N1 and N2P2-amplitudes were square root transformed to improve normality of residuals and coefficient magnitudes should be interpreted accordingly. The constant represents first-block amplitude or pain score responses in controls. The main effect of migraine represents the first-block amplitude or pain score difference between groups. The main effect of habituation represents the difference between first and second block amplitudes, or linear change in pain scores, in the control group. The interaction effect represents the habituation-difference between groups. Thus, the significant coefficient is interpreted as N2P2 habituation in the control group. The corresponding interaction effect is not significant, indicating no difference in habituation between controls and interictal migraineurs. Random effect estimates are displayed as variances. * *p* < 0.05, ** *p* < 0.01, *** *p* < 0.001.

**Table S4.** Estimated amplitudes and habituation of N1 and N2P2 by phase and the effect of aura.

|  | **N1 (µV^0.5^)** | |  | **N2P2 (µV^0.5^)** | |
| --- | --- | --- | --- | --- | --- |
|  | **Coef.** | **95% CI** |  | **Coef.** | **95% CI** |
| **Main effects** |  |  |  |  |  |
| Preictal | 0.188 | [-0.142, 0.518] |  | 0.245*** | [-0.116, 0.605] |
| Ictal | -0.193 | [-0.488, 0.102] |  | 0.074*** | [-0.348, 0.497] |
| Postictal | 0.031 | [-0.228, 0.291] |  | 0.145*** | [-0.336, 0.627] |
|  |  |  |  |  |  |
| Habituation | -0.065 | [-0.197, 0.067] |  | -0.286*** | [-0.445, -0.128] |
|  |  |  |  |  |  |
| Aura | 0.199 | [-0.131, 0.530] |  | 0.218*** | [-0.439, 0.876] |
| **Two-way interaction effects** |  |  |  |  |  |
| Preictal × Habituation | -0.201 | [-0.555, 0.152] |  | 0.038*** | [-0.287, 0.364] |
| Ictal × Habituation | 0.102 | [-0.178, 0.382] |  | 0.000*** | [-0.400, 0.400] |
| Postictal × Habituation | -0.124 | [-0.439, 0.192] |  | 0.046*** | [-0.402, 0.494] |
|  |  |  |  |  |  |
| Preictal × Aura | -0.063 | [-0.578, 0.451] |  | -0.324*** | [-0.875, 0.228] |
| Ictal × Aura | 0.134 | [-0.263, 0.531] |  | -0.021*** | [-0.677, 0.636] |
| Postictal × Aura | 0.273 | [-0.180, 0.726] |  | 0.177*** | [-0.639, 0.993] |
|  |  |  |  |  |  |
| Habituation × Aura | -0.174 | [-0.448, 0.099] |  | -0.060*** | [-0.333, 0.212] |
| **Three-way interaction effects** |  |  |  |  |  |
| Preictal × Habituation × Aura | 0.137 | [-0.391, 0.665] |  | -0.153*** | [-0.660, 0.353] |
| Ictal × Habituation × Aura | -0.077 | [-0.444, 0.291] |  | -0.067*** | [-0.691, 0.557] |
| Postictal × Habituation × Aura | -0.161 | [-0.928, 0.607] |  | 0.061*** | [-0.696, 0.819] |
|  |  |  |  |  |  |
| **Constant** | 2.592 | [2.370, 2.814] |  | 5.989*** | [5.595, 6.382] |
|  |  |  |  |  |  |
|  | **Estimate** | |  | **Estimate** | |
| **Random effects** |  |  |  |  |  |
| Level 3: subject | 0.173 | |  | 1.042 | |
| Level 2: session | 0.046 | |  | 0.166 | |
| Level 1: residuals | 0.152 | |  | 0.211 | |

N1 and N2P2-amplitudes were square root transformed to improve normality of residuals and coefficient magnitudes should be interpreted accordingly. The constant represents interictal first-block responses in subjects with migraine without aura. None of the three-way interactions were significant, that is, habituation-differences between phases were not different between migraineurs with and without aura. Interictal first-block amplitudes and habituation were not different between migraineur with and without aura. Random effect estimates are displayed as variances. * *p* < 0.05, ** *p* < 0.01, *** *p* < 0.001.

**Table S5.** Estimated amplitudes and habituation of N1 and N2P2 by phase and the effect of headache laterality.

|  | **N1 (µV^0.5^)** | |  | **N2P2 (µV^0.5^)** | |
| --- | --- | --- | --- | --- | --- |
|  | **Coef.** | **95% CI** |  | **Coef.** | **95% CI** |
| **Main effects** |  |  |  |  |  |
| Preictal | 0.043 | [-0.419, 0.505] |  | 0.119** | [-0.425, 0.662] |
| Ictal | -0.085 | [-0.473, 0.303] |  | 0.355** | [-0.280, 0.990] |
| Postictal | -0.063 | [-0.692, 0.567] |  | 0.562** | [-0.384, 1.508] |
|  |  |  |  |  |  |
| Habituation | -0.028 | [-0.221, 0.165] |  | -0.336** | [-0.551, -0.120] |
|  |  |  |  |  |  |
| Left side | 0.121 | [-0.222, 0.464] |  | -0.001** | [-0.538, 0.536] |
| Bilateral | -0.152 | [-0.698, 0.393] |  | 0.161** | [-0.583, 0.905] |
| **Two-way interaction effects** |  |  |  |  |  |
| Preictal × Habituation | -0.150 | [-0.612, 0.313] |  | 0.077** | [-0.355, 0.509] |
| Ictal × Habituation | -0.195 | [-0.459, 0.069] |  | -0.164** | [-0.671, 0.343] |
| Postictal × Habituation | -0.231 | [-0.998, 0.537] |  | -0.292** | [-1.072, 0.487] |
|  |  |  |  |  |  |
| Preictal × Left side | 0.162 | [-0.549, 0.873] |  | -0.224** | [-1.185, 0.737] |
| Preictal × Bilateral | 0.342 | [-0.502, 1.187] |  | 0.084** | [-0.784, 0.952] |
| Ictal × Left side | -0.031 | [-0.617, 0.555] |  | -0.029** | [-1.132, 1.073] |
| Ictal × Bilateral | -0.051 | [-0.684, 0.581] |  | -0.886** | [-1.939, 0.167] |
| Postictal × Left side | 0.245 | [-0.407, 0.898] |  | -0.196** | [-1.346, 0.955] |
| Postictal × Bilateral | 0.427 | [-0.445, 1.298] |  | -0.764** | [-2.052, 0.523] |
|  |  |  |  |  |  |
| Habituation × Left side | -0.170 | [-0.429, 0.089] |  | 0.113** | [-0.193, 0.418] |
| Habituation × Bilateral | 0.168 | [-0.300, 0.636] |  | -0.356** | [-0.835, 0.123] |
| **Three-way interaction effects** |  |  |  |  |  |
| Preictal × Habituation × Left side | -0.012 | [-0.516, 0.491] |  | -0.087** | [-0.682, 0.508] |
| Preictal × Habituation × Bilateral | -0.283 | [-1.209, 0.644] |  | 0.016** | [-0.718, 0.749] |
| Ictal × Habituation × Left side | 0.344 | [-0.170, 0.857] |  | -0.098** | [-0.862, 0.667] |
| Ictal × Habituation × Bilateral | 0.143 | [-0.440, 0.727] |  | 0.822** | [-0.003, 1.647] |
| Postictal × Habituation × Left side | -0.074 | [-0.966, 0.819] |  | 0.121** | [-0.909, 1.150] |
| Postictal × Habituation × Bilateral | -0.174 | [-1.288, 0.940] |  | 1.029** | [0.014, 2.045] |
|  |  |  |  |  |  |
| **Constant** | 2.644 | [2.367, 2.921] |  | -0.814** | [-1.046, -0.582] |
|  |  |  |  |  |  |
|  | **Estimate** | |  | **Estimate** | |
| **Random effects** |  |  |  |  |  |
| Level 3: subject | 0.198 | |  | 0.998 | |
| Level 2: session | 0.044 | |  | 0.196 | |
| Level 1: residuals | 0.144 | |  | 0.219 | |

N1 and N2P2-amplitudes were square root transformed to improve normality of residuals and coefficient magnitudes should be interpreted accordingly. Headache laterality was classified by the related attack if the phase was preictal, ictal or postictal. Interictal recordings were classified by the side the subject most commonly experienced headache, either left, right or bilateral. Sixteen interictal recordings had an equal amount of left and right-sided unilateral migraine and were not included in this analysis. The constant represents interictal first-block responses in subjects with right-sided migraine, the same side as the laser-stimuli were applied. Subjects with bilateral migraine had reduced N2P2-habituation (more positive slope) in the postictal phase compared to the interictal phase, and the same tendency was present in the ictal phase. Habituation was not different between left and right-sided migraine. Headache laterality did not significantly affect interictal estimates of first-block amplitude and habituation. Random effect estimates are displayed as variances. * *p* < 0.05, ** *p* < 0.01, *** *p* < 0.001.

**Table S6.** Estimated amplitudes and habituation of N1 and N2P2 by phase and the effect of years lived with migraine (YwM).

|  | **N1 (µV^0.5^)** | |  | **N2P2 (µV^0.5^)** | |
| --- | --- | --- | --- | --- | --- |
|  | **Coef.** | **95% CI** |  | **Coef.** | **95% CI** |
| **Main effects** |  |  |  |  |  |
| Preictal | 0.136* | [-0.092, 0.365] |  | 0.082*** | [-0.197, 0.362] |
| Ictal | -0.124* | [-0.341, 0.092] |  | 0.098*** | [-0.223, 0.419] |
| Postictal | 0.082* | [-0.157, 0.320] |  | 0.141*** | [-0.252, 0.533] |
|  |  |  |  |  |  |
| Habituation | -0.124* | [-0.246, -0.002] |  | -0.307*** | [-0.433, -0.180] |
|  |  |  |  |  |  |
| YwM | -0.016* | [-0.034, 0.003] |  | -0.002*** | [-0.039, 0.036] |
| **Two-way interaction effects** |  |  |  |  |  |
| Preictal × Habituation | -0.145* | [-0.394, 0.104] |  | -0.013*** | [-0.257, 0.231] |
| Ictal × Habituation | 0.053* | [-0.149, 0.255] |  | -0.042*** | [-0.343, 0.259] |
| Postictal × Habituation | -0.120* | [-0.407, 0.167] |  | 0.103*** | [-0.259, 0.464] |
|  |  |  |  |  |  |
| Preictal × YwM | -0.012* | [-0.046, 0.022] |  | -0.020*** | [-0.056, 0.016] |
| Ictal × YwM | 0.007* | [-0.011, 0.025] |  | 0.025*** | [-0.009, 0.058] |
| Postictal × YwM | -0.012* | [-0.052, 0.028] |  | -0.023*** | [-0.068, 0.022] |
|  |  |  |  | *** |  |
| Habituation × YwM | 0.005* | [-0.010, 0.020] |  | -0.005*** | [-0.018, 0.008] |
| **Three-way interaction effects** |  |  |  |  |  |
| Preictal × Habituation × YwM | 0.009* | [-0.018, 0.036] |  | 0.037*** | [0.009, 0.066] |
| Ictal × Habituation × YwM | -0.011* | [-0.028, 0.006] |  | 0.014*** | [-0.017, 0.046] |
| Postictal × Habituation × YwM | 0.029* | [-0.027, 0.085] |  | 0.024*** | [-0.019, 0.066] |
| **Control variables** |  |  |  |  |  |
| Age | 0.000* | [-0.014, 0.014] |  | -0.038*** | [-0.070, -0.005] |
| Migraine intensity 1 | -0.525* | [-1.082, 0.032] |  | -0.539*** | [-2.093, 1.015] |
| Migraine intensity 3 | -0.186* | [-0.413, 0.042] |  | -0.336*** | [-0.942, 0.269] |
| Migraine frequency 1 | 0.130* | [-0.152, 0.413] |  | 0.130*** | [-0.529, 0.789] |
| Migraine frequency 3 | 0.398* | [0.049, 0.747] |  | 0.980*** | [-0.025, 1.986] |
|  |  |  |  |  |  |
| **Constant** | 2.715* | [2.481, 2.949] |  | 6.142*** | [5.662, 6.621] |
|  |  |  |  |  |  |
|  | **Estimate** | |  | **Estimate** | |
| **Random effects** |  |  |  |  |  |
| Level 3: subject | 0.139 | |  | 0.898 | |
| Level 2: session | 0.046 | |  | 0.167 | |
| Level 1: residuals | 0.152 | |  | 0.203 | |

N1 and N2P2-amplitudes were square root transformed to improve normality of residuals and coefficient magnitudes should be interpreted accordingly. Years with migraine (YwM) and age were centered at their means. Migraine intensity and frequency were dummy-coded with category 2 as base in both, i.e. moderate intensity and 4-7 days with migraine/month (intensity: 1: mild, 2: moderate, 3: severe, and frequency: 1: 1-3 days/month, 2: 4-7 days/month, 3: 8-14 days/month). The constant represents interictal first-block responses at mean YwM, mean age, migraine intensity = 2, and migraine frequency = 2. Subjects who have lived more years with migraine have decreased N2P2-habituation in the preictal phase compared to the interictal phase. First-block amplitude and habituation in the interictal phase did not differ with YwM. Random effect estimates are displayed as variances. * *p* < 0.05, ** *p* < 0.01, *** *p* < 0.001.

**Table S7.** Estimated amplitudes and habituation of N1 and N2P2 by phase and the effect of pain scores.

|  | **N1 (µV^0.5^)** | |  | **N2P2 (µV^0.5^)** | |
| --- | --- | --- | --- | --- | --- |
|  | **Coef.** | **95% CI** |  | **Coef.** | **95% CI** |
| **Main effects** |  |  |  |  |  |
| Preictal | 0.195* | [-0.045, 0.435] |  | 0.134*** | [-0.116, 0.384] |
| Ictal | -0.138* | [-0.338, 0.063] |  | 0.091*** | [-0.206, 0.388] |
| Postictal | 0.157* | [-0.035, 0.348] |  | 0.121*** | [-0.270, 0.512] |
|  |  |  |  |  |  |
| Habituation | -0.129* | [-0.252, -0.006] |  | -0.312*** | [-0.435, -0.188] |
|  |  |  |  |  |  |
| Pain scores | 0.092* | [0.014, 0.170] |  | 0.219*** | [0.142, 0.295] |
| **Two-way interaction effects** |  |  |  |  |  |
| Preictal × Habituation | -0.170* | [-0.417, 0.077] |  | -0.084*** | [-0.324, 0.156] |
| Ictal × Habituation | 0.068* | [-0.142, 0.277] |  | -0.051*** | [-0.350, 0.247] |
| Postictal × Habituation | -0.210* | [-0.506, 0.087] |  | -0.117*** | [-0.512, 0.278] |
|  |  |  |  |  |  |
| Preictal × Pain scores | -0.024* | [-0.131, 0.083] |  | -0.031*** | [-0.159, 0.097] |
| Ictal × Pain scores | 0.014* | [-0.095, 0.122] |  | 0.010*** | [-0.149, 0.169] |
| Postictal × Pain scores | -0.020* | [-0.120, 0.081] |  | 0.003*** | [-0.141, 0.146] |
|  |  |  |  |  |  |
| Habituation × Pain scores | -0.005* | [-0.065, 0.056] |  | -0.028*** | [-0.088, 0.032] |
| **Three-way interaction effects** |  |  |  |  |  |
| Preictal × Habituation × Pain scores | -0.018* | [-0.152, 0.116] |  | 0.009*** | [-0.119, 0.137] |
| Ictal × Habituation × Pain scores | -0.063* | [-0.172, 0.046] |  | -0.073*** | [-0.225, 0.079] |
| Postictal × Habituation × Pain scores | -0.008* | [-0.165, 0.150] |  | 0.089*** | [-0.052, 0.229] |
|  |  |  |  |  |  |
| **Constant** | 2.652* | [2.486, 2.817] |  | 6.039*** | [5.719, 6.359] |
|  |  |  |  |  |  |
|  | **Estimate** | |  | **Estimate** | |
| **Random effects** |  |  |  |  |  |
| Level 3: subject | 0.173 | |  | 1.118 | |
| Level 2: session | 0.037 | |  | 0.119 | |
| Level 1: residuals | 0.151 | |  | 0.193 | |

N1 and N2P2-amplitudes were square root transformed to improve normality of residuals and coefficient magnitudes should be interpreted accordingly. Pain scores were measured by a numerical rating scale (NRS) ranging from 0 = no pain to 10 = unbearable pain. Pain scores were centered at NRS = 4 before analysis. Thus, the constant represents interictal first-block responses at NRS = 4. There were no phase-differences in habituation by pain scores (none of the three-way interactions were significant). Pain scores did not affect habituation in the interictal phase, as shown by the “Habituation × Pain scores” interaction. Interictal N1 and N2P2 first-block amplitudes increased with increasing pain scores, as shown by the main effects of pain scores. Random effect estimates are displayed as variances. * *p* < 0.05, ** *p* < 0.01, *** *p* < 0.001.
